# Generation of Individualized Synthetic Data for Augmentation of the Type 1 Diabetes Data Sets Using Deep Learning Models

**DOI:** 10.3390/s22134944

**Published:** 2022-06-30

**Authors:** Josep Noguer, Ivan Contreras, Omer Mujahid, Aleix Beneyto, Josep Vehi

**Affiliations:** 1 Institut d’Informàtica i Aplicacions, Universitat de Girona, 17003 Girona, Spain; jnoguertorres@gmail.com (J.N.); ivan.contreras@udg.edu (I.C.); omer.mujahid@udg.edu (O.M.); aleix.beneyto@udg.edu (A.B.); 2 Centro de Investigación Biomédica en Red de Diabetes y Enfermedades Metabólicas Asociadas (CIBERDEM), 28029 Madrid, Spain

**Keywords:** deep learning, blood glucose, generative model, artificial intelligence, type 1 diabetes

## Abstract

In this paper, we present a methodology based on generative adversarial network architecture to generate synthetic data sets with the intention of augmenting continuous glucose monitor data from individual patients. We use these synthetic data with the aim of improving the overall performance of prediction models based on machine learning techniques. Experiments were performed on two cohorts of patients suffering from type 1 diabetes mellitus with significant differences in their clinical outcomes. In the first contribution, we have demonstrated that the chosen methodology is able to replicate the intrinsic characteristics of individual patients following the statistical distributions of the original data. Next, a second contribution demonstrates the potential of synthetic data to improve the performance of machine learning approaches by testing and comparing different prediction models for the problem of predicting nocturnal hypoglycemic events in type 1 diabetic patients. The results obtained for both generative and predictive models are quite encouraging and set a precedent in the use of generative techniques to train new machine learning models.

## 1. Introduction

In the past few decades, machine learning techniques have enabled the development of powerful systems in a wide range of application areas, turning data acquisition into a top priority. The development of applications for the management of type 1 diabetes mellitus (T1DM), where the use of data-based models has experienced important growth [1], has witnessed the consequences of this phenomenon. Data acquisition related to T1DM clinical studies and commercial applications has been simplified by technological advances. One of the most significant advances has been the generalization of continuous glucose monitors (CGM), which have the ability to continuously capture blood glucose levels. Despite this, most of the data collected are not freely available and tend to be heterogeneous due to the variety of factors and scenarios that affect T1DM patients [2], which hinders obtaining a sufficient number of samples or information for the specific objectives to be addressed. In addition, most of the applications developed in this field require information on other main disturbances, commonly related to insulin doses, meals, or physical activity [3,4], which are typically recorded manually by patients, making these data prone to human error. Finally, another key problem appears with data protection legislation and although there are initiatives [5,6] that release anonymous data for the use in research, data collection and processing are often associated with complex bureaucratic procedures and expensive clinical trials, becoming even more complex when dealing with data exchanges between different countries.

This study aims to contribute by:

1. Proposing a method to expand series of continuous blood glucose measurement at the individual level.
2. Devising a nocturnal hypoglycemia classifier based on a data-augmented approach.

The first contribution is the implementation of a deep learning model capable of generating realistic CGM sequences; the second contribution is the comparison of the performance of a nocturnal hypoglycemia predictor using or not using augmented data. The generated time series are intended to replicate the intrinsic characteristics of specific individuals with T1DM without explicitly duplicating values and will be evaluated using clinical outcomes and statistically based metrics. The synthetic data generator is driven by the demand for competitive predictive models based on restricted data resources; therefore, it can provide a solution to persistent data shortage, i.e., cases where it is difficult to obtain more samples, such as small and highly specific populations or with high collection costs; it can also solve the problem of time-related data shortage, i.e., cases where answers are required from the early stages of data collection. This proposal could be useful in a varied range of clinical scenarios, although it can be implemented in any approach that uses blood glucose to build models. The nocturnal hypoglycemia classifier intends to demonstrate the advantages of using synthetic data in the field of nocturnal hypoglycemia prediction, where the data sets used for model training are small and highly imbalanced. The improved models aim to improve prediction performance under unfavorable conditions and with only CGM data available. These models are designed to help find a solution to predict nocturnal hypoglycemia. As a decision support system, it intends to prevent patients from suffering from a severe decrease in blood glucose, by allowing them to act accordingly to the prediction, for example, in-taking a small quantity of carbohydrates.

Furthermore, an additional contribution of this proposed method lies in its ability to extract underlying patterns from these data, which can be applied for sample anonymization purposes, potentially making the data sets more open to be shared, as they would be excluded from data protection legislation.

### 1.1. State-of-the-Art

Nowadays, the application of artificial intelligence AI to the field of diabetes management has a long track of progress [7]. Unfortunately, most AI-based applications are data-dependent approaches that often suffer from the limitations caused by the lack of quantity and quality of data sets. This is also reflected in modeling approaches, which tend to target the population level; therefore, there are few practical examples of models on the individualized level [8], limiting the possibilities and contributing to slowing down the progress in different fields of applications of these techniques [9].

To meet the challenge of data collection and human trials, the so-called virtual simulators were introduced to validate new treatments in silico and generate synthetic data. Particularly in the area of diabetes, we can find numerous software that provide tools to generate virtual patients, mostly used in the validation of control algorithms. These software witnessed a great leap forward after 2008, when the FDA allowed the use of one such T1D simulator in pre-clinical trials of some insulin treatments [10]. These simulators follow specific mathematical functions that relate carbohydrate consumption and insulin administration to generate new blood glucose time series. The formulas used are specifically designed to replicate the behavior of human metabolism; however, one of the main problems in these tools is that, as they respond to mathematical functions, they do not accurately represent real samples. This problem is commonly referenced as the reality gap problem [11], which complicates the use of artificial data for deep learning.

Many deep learning proposals have appeared in order to fill this lack of data and avoid the reality gap problem. For example, in the field of image analysis, generative adversarial networks (GAN) are used to enlarge a small set of images with synthetic data, for a posterior use in classification problems, with results demonstrating its effectiveness [12,13]. A similar case is found in electrocardiogram (EKG) generation [14], where the authors present a GAN with the capacity of producing realistically EKG, valid to train machine learning applications. In addition, in the field of cardiology and with the intention of data anonymization many articles use adaptations of image generation GAN to produce realistic samples [15,16]. In the specific field of using synthetic data for glucose prediction, the article [17] proposes a model that implements different data augmentation techniques to balance data sets and improve the performance of a glucose prediction model for patients with type 2 diabetes mellitus at different time horizons, achieving state-of-the-art results.

### 1.2. Diabetes Mellitus

T1DM is a chronic pathology involving the destruction of pancreas β cells and consequently producing an insulin deficit [18]. Insulin is a peptidic hormone necessary for blood glucose regulation and maintenance of homeostasis [19] favoring its transportation into organs and muscular tissues. T1DM patients require the administration of external doses of insulin to maintain stable blood glucose levels. Some of the risks of not properly controlling these levels are translated into a possible excess or lack of blood glucose, called hyperglycemia (Hyper) and hypoglycemia, respectively. Hyperglycemia is defined as glucose values above 180 mg/dL and its main risks are suffering from diabetic ketoacidosis or a hyperosmolar hyperglycemic state, which has important long-term complications. A patient with hypoglycemia can suffer from neurological dysfunction including mild impairments (dizziness, somnolence) to severe conditions such as coma and death [19]. The effects of hypoglycemia vary depending on its intensity. A level 1 hypoglycemia (L1 Hypo) is a decrease in blood glucose levels under 70 mg/dL, while a level 2 hypoglycemia (L2 Hypo) is a drop in blood glucose levels below 54 mg/dL. The time period of blood glucose between 70 mg/dL and 180 mg/dL is named as time in range TIR [20].

## 2. Materials and Methods

### 2.1. Data Collection and Preprocessing

We have targeted two data sources corresponding to different T1DM cohorts. The first cohort corresponds to patients from the observational trial of Bertachi et al. [21] carried out at the hospital clinic of Barcelona. Blood glucose measurements were collected from CGM of ten adults suffering from T1DM that were studied for 12 weeks under home free living conditions. The data set involves individuals prone to hypoglycemia, defined as those patients with more than 4 hypoglycemias per week. The other data source corresponds to the Ohio T1DM data set [5] and involves measurements up to 8 weeks of blood glucose data of 6 adult patients with T1DM wearing a Medtronic 530G insulin pump and using Medtronic Enlite CGM sensors.

A series of data preprocessing procedures have been applied to obtain consistent data sets. First of all, an exploratory analysis pointed out missing CGM measurements. Missing data have been found both in short (less than 1 h) and in long periods. Generally, in the short periods, they are due to a punctual loss of signal, and in the longer periods, they are related to battery problems, sensor replacement, software problems, etc., which require a longer response time to solve. Thus, missing values between two measurements separated by less than 1h have been linearly interpolated. Then, we have extracted time series to comply with the purposes of this study. This has been performed for every patient individually obtaining a different data set for every patient. These data sets are built into the form of a matrix with 288 columns, each column representing the glucose at 5 min interval.

### 2.2. Generative Adversarial Networks

The synthetic data used to augment the original data sets have been generated using a neural network scheme known as GAN, with a convolutional-based structure. This type of GAN architecture has shown great success in generating images, video, and temporal data [22], being able to perform better than recurrent neural networks in some cases [23]. Defined in 2014 [24], this type of neural network combines two smaller networks that are trained in an orderly manner. These networks are named generator and discriminator. The first one produces samples that try to resemble real data from a random probability distribution named latent space. The discriminator is trained to differentiate between real and synthetic samples. The GAN training method consists of a MinMax optimizer where the discriminator tries to reduce its error and the generator that tries to maximize it, which makes it able to generate more realistic samples over time.

**Figure 1:**
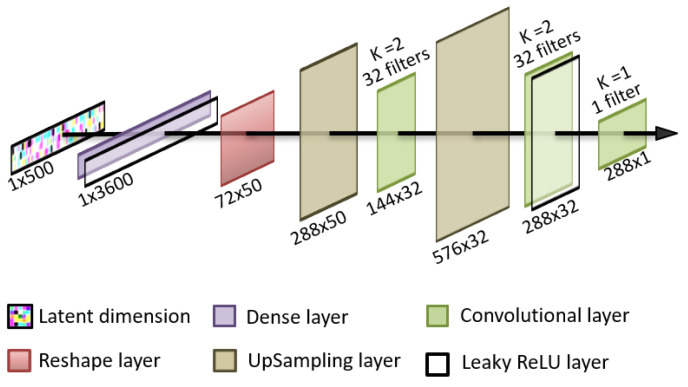
Graphical representation of the generator architecture.

The discriminator and the generator are neural networks with distinct architectures. The method chosen in this paper is a unidimensional adaptation of image generation GAN for both the generator and the discriminator by using convolutional layers. The specific structure of the generator uses, as latent space, random noise from the normal distribution. The latent space is introduced into an input dense layer capable of representing 50 versions of the same low definition sequence. These 50 versions are then sequentially upsampled and convolutionally filtered to generate an output sequence consisting in 288 values of blood glucose that represent a single day of a patient. This architecture is shown in Figure 1.

**Figure 2:**
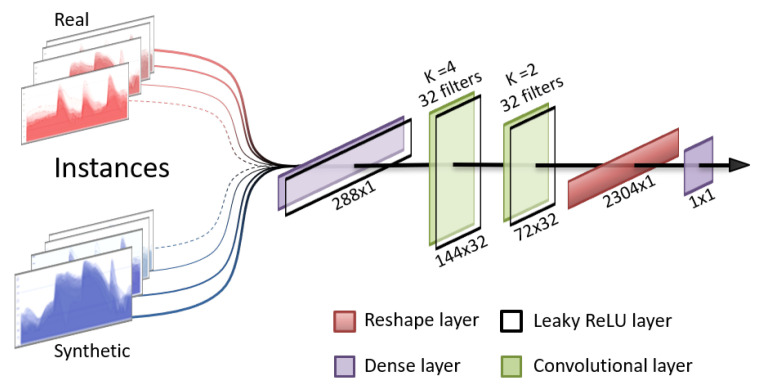
Graphical representation of the discriminator architecture.

For the discriminator, the chosen structure is an adaptation of image classification models, consisting of a sequence of convolutions and leaky rectified linear units (ReLU) layers and has an output layer of a single neuron with a sigmoid activation function. This forces the discriminator to produce output values of 0 or 1, representing “synthetic” and “real”, respectively. The structure of the discriminator is presented in Figure 2.

As the discriminator is trained to distinguish between real samples with a specific temporal frame and synthetic samples, it has the ability to associate as “not real”, the sequences which do not belong to the specific window; therefore, the overall GAN cannot produce rolling windows of the original data.

### 2.3. Augmented Data-Based Nocturnal Hypoglycemia Predictor

As a real application of augmented data, we propose to use it as a tool to extend imbalanced data sets with few instances. To this end, the approach is to use deep generative models to increase the number of training instances in the context of a nocturnal hypoglycemia classifier.

**Figure 3:**
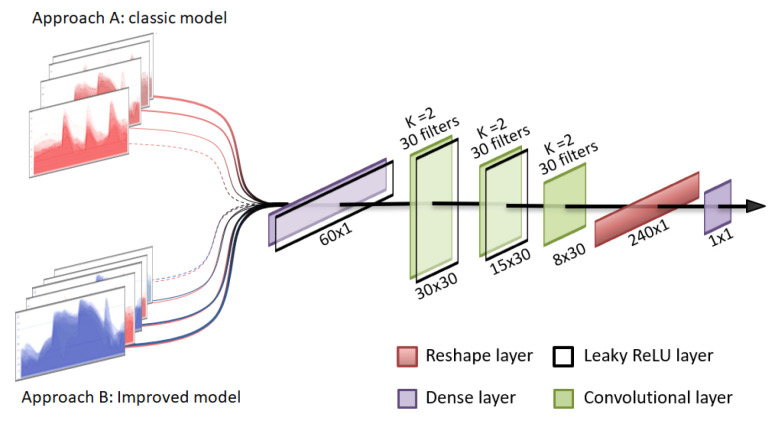
Graphical representation of the nocturnal hypoglycemia classifier.

A uni-dimensional convolutional classifier has been implemented to compare the performances of prediction models trained with the original data and an extended version of these data sets using generated synthetic data, hereafter referenced as the augmented data sets. The architecture of the implemented methodology is presented in Figure 3 and is similar to the one used for the discriminator in the GAN model. This type of artificial neural network has demonstrated great success in classification problems and also in the specific case of glucose prediction [25]. It has been specially designed to classify days into two categories, depending on whether the patients have nocturnal hypoglycemia or not.

Nocturnal hypoglycemia events are defined when the continuous glucose monitor registers three consecutive values below 70 mg/dL in the time period between 10:00 p.m. and 06:00 a.m. The implemented system makes the predictions using only the CGM values prior to this time period, specifically with the CGM measurements of the previous 5 h, between 5:00 p.m. and 10:00 p.m. The information recorded after 10:00 p.m. is exclusively used to label the instances into nights with hypoglycemia and nights without hypoglycemia to either train the algorithm or evaluate the performance of the algorithm. Since nocturnal hypoglycemia is most often than not the cumulative effect of all the decisions we take during the day and the blood glucose values prior to a patient’s sleeping hours have a major impact on the occurrence of nocturnal hypoglycemia, we harness the impact of this fact in the hypoglycemia predictor. The premise is that at the time of making the prediction, the application should be able to detect whether the patient is going to have nocturnal hypoglycemia or not and provide patients the opportunity to take the appropriate measures to avoid it. After processing the data sets, the number of total instances available for each patient are presented in Table 1 for the Barcelona cohort and in Table 2 for the Ohio cohort. It can be noticed that the number of nocturnal hypoglycemia events is considerably lower in the Ohio data set as well as the number of days. Stratified k-Fold cross-validation has been used to evaluate the models. It should be noted that only non-synthetic data sets were used as testing data.

### 2.4. Evaluation Mechanisms

To assess the performance of the generative model and the real implications of this method in glucose prediction examples, we have used different evaluation metrics. First, real and synthetic data have been evaluated and compared using time-in-range metrics, which measure the percentage of time that blood glucose is in certain ranges. These metrics are considered standard clinical outcomes in patients with diabetes and, in our case, will determine whether the generated patients are clinically similar to real patients. We have accepted the generative models when they match the specific values of the real data sets used to train the model via a Wilcoxon test. This test is built to determine if there are differences between two paired groups, meaning a *p*-value over 0.05 shows a statistical significance between the two samples. We have also compared other important statistical metrics which are mean blood glucose, standard deviation (SD) and variance, Jensen–Shannon (JS) distance, and Z-Test, which provide relevant information about the distributions.

JS divergence is an application of Kullback–Leibler divergence and shows the degree of similarity between two probability distributions [26] or temporal series [27]. In contrast with the Kullback–Leibler divergence, this can have values only between 0 and 1; 0 being representative of two identical distributions. We have used the square root of Jensen–Shannon JS divergence, which is named JS distance, calculated with Equation (1)

(1) $$\begin{array}{c} JSd=\sqrt{\frac{D(P∥M)+D(Q∥M)}{2}} \end{array}$$

where M is defined as $M=\frac{1}{2}\left(P+Q\right)$.

For statistical metrics that relate a distribution with another (JS, Z-test, variance) we have evaluated the means of obtained values when comparing real–synthetic, real–real, and synthetic–synthetic samples. This provides a global vision of the expected values for each metric. For example, if JS distance is equal to 0.50 in the real–real test, the expected value in the synthetic–synthetic and in the real–synthetic test is also 0.50. Moreover, JS heat maps have been presented to demonstrate that the generative model does not replicate the original samples. They are expected to have dark blue tones, representing identical comparisons only when comparing samples against themselves and green tones for the remaining comparisons.

Additionally, to evaluate the GAN model, we have used different ranges of values, studied for each day of the real data set, compared to each sample of the synthetic one. The different threshold values for each range are represented in Table 3.

To validate the nocturnal hypoglycemia classifier, the metrics of accuracy (ACC), sensitivity (SEN), specificity (SP), Matthews correlation coefficient (MCC), and geometric mean (G${}_{mean}$), defined as $\sqrt{\mathrm{SEN}\times \mathrm{SP}}$, have been used.

### 2.5. Technical Specifications

For all the coding in this paper, a Python-based program has been used. Models have been programmed with Keras [28] and TensorFlow [29]. For calculations and data processing, we have used Pandas [30] and Numpy [31]; to calculate the performance metrics and graphical visualization, the scikit-learn [32] and Matplotlib [33] packages have been used.

The hardware specifications are presented in Table 4.

## 3. Results

In this section, we present the results obtained for both the generative and the classification model. We produced a total of 10 synthetic data sets for each patient of the original Barcelona cohort and 6 data sets for each patient of the Ohio cohort. The obtained scores, when individually comparing synthetic and real patients, are presented in Table 5 and Table 6 for the Barcelona and Ohio data sets, respectively. The metrics are shown for each batch of days, taking for the synthetic patients a number of samples equal to their respective amount of days as the real ones. We have studied the different times in range and the mean glucose value by comparing the real and synthetic data sets and obtaining a *p*-value via the Wilcoxon test. It is important to notice that this test accepts, as different distributions, the ones that produce *p*-values under 0.05. The obtained results are over this threshold for every patient so they have been considered as not different and thus valid.

**Figure 4:**
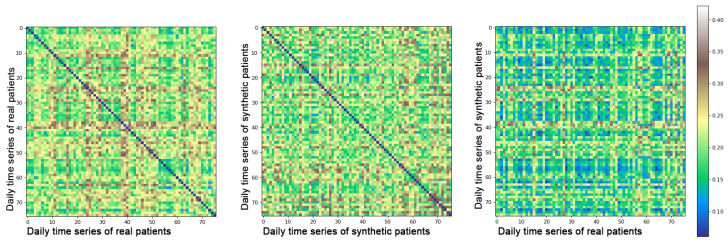
Heat maps representing distances of real and synthetic samples of Barcelona patients.

Heat maps with the JS comparison are included in Figure 4 as an exemplification of the models performance when it comes to evaluating how original the synthetic data are. It can be observed that when comparing real–real samples, JS values of 0 appear represented in dark blue on the heat map, meaning that those samples are identical. To identify how similar generated samples are between themselves, we have studied the second heat map of the figure. We can notice that there are no repeated samples, as no dark blue values are represented out of the diagonal. To prove that the proposed method does not identically replicate the original data, the last heat map shows a comparison of real–synthetic data, and as to whether all values tend to be lower than in the real–real comparison, no identical (zero value) comparisons appear. We present all three heat maps only with samples of patient number 1 of the Barcelona data as an example, although these heatmaps present similar patterns for each of the individual patients used in the study.

**Figure 5:**
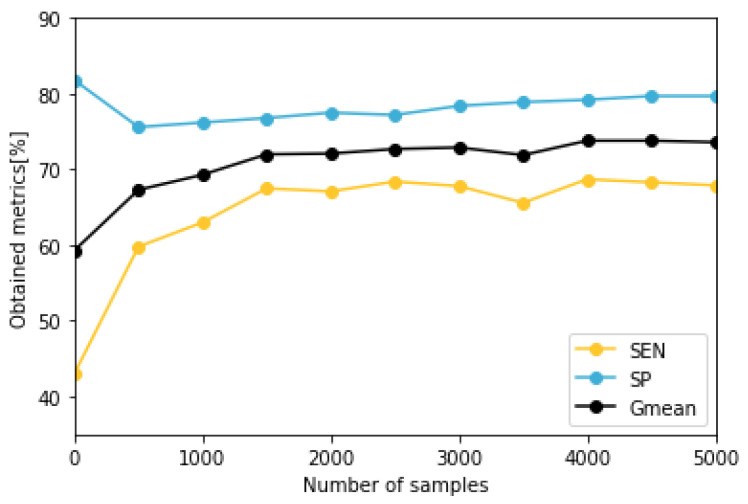
Representation of the obtained metrics related to the amount of days used to augment the real data set.

To train and evaluate the predictive model, patients with less than five nocturnal hypoglycemic events (B4, O1, O3, and O5) have been discarded as the reduced amount of events is an important methodological limitation and makes testing data unsuitable to evaluate the classifier accurately. The models corresponding to the remaining patients have been validated with a stratified k-Fold cross validation method, using for each fold different train and test data. To determine the optimal number of days to use in the augmented data set, we have calculated the median metrics of the models trained with different number of synthetic samples. We have generated data sets of between 500 and 5000 instances and trained the predictive model 20 times for each patient and number of synthetic days. We have observed that the results improve with a higher number of samples, although for values higher than 1500 synthetic samples, the increase is slow, as presented in Figure 5.

Table 7 presents in detail the results obtained when using 5000 days of synthetic data for training the models in patients for both cohorts. The models were evaluated in terms of ACC, SEN, SP, MCC, and G${}_{mean}$. Results are presented only using real data to test the model, as the purpose of the testing is to demonstrate that augmented data can represent real life events. The achieved outcomes show a general improvement in the majority of the metrics and for every patient of both data sets.

## 4. Discussion

The obtained results, presented in Table 5 and Table 6, demonstrate that each synthetic data set correlates with its real counterpart in terms of the variables used to numerically describe each patient. The obtained CGM values follow the same time distributions of Hyper, TIR, L1 Hypo, and L2 Hypo of the original data and additionally correspond with the other values of mean, JS, variance, Z-Value, and SD. These results prove that the proposed GAN architecture is valid for generating new time series of blood glucose values. The presented outcomes for the different statistics show that each synthetic data set is a valid representation of its real counterpart. As the different tests have demonstrated in terms of times in range, mean, and other measurements, the GAN model has been able to represent into synthetic data the intrinsic characteristics of the original one. This implementation is an advantage for machine learning development and implies an advancement to reduce the reality gap problem. Since the methodology relies on a mathematical function designed by the machine, the generated data sets are able to respond more accurately to a realistic behavior. This fact combined with the possibility of augmenting real data sets indeterminately and with a very small computational cost, implies that the usage of GAN has potential to become a common practice when developing machine learning applications.

The nocturnal hypoglycemia classifier trained with both real and augmented data sets, has demonstrated that using synthetic data to expand reduced and imbalanced data sets is a valid method to use in machine learning applications. The experiments conducted in this study have shown that for every patient included in this trial there has been an improvement in terms of some of the most common evaluation metrics (ACC, SEN, SP, MCC, and $G_{mean}$) showing a percentage of improvement of 11.5% for the mean of all the metrics and every patient. In the specific problem of predicting nocturnal hypoglycemia, due to the strong unbalance in data, the most relevant metrics are SP and SEN. High scores in the first one corresponds to correctly predicting most of the nocturnal hypoglycemia events and elevated results; the second implies a correct classification of nights with no hypoglycemia and therefore not encouraging the patient to take measures that could increase the amount of blood glucose unnecessarily. In the results from Table 7 show that SEN presents an important improvement when the values of SP are slightly reduced. As the metrics of $G_{mean}$ and MCC are a balanced representation of the combined SEN and SP, their improvement is translated into a more reliable model.

Another key aspect to consider is the fact that the prediction is performed only with blood glucose data and not using information related to insulin, carbohydrate intake, or other important disturbances such as physical exercise, which are often used in the nocturnal classification problem [34]. Despite this restriction, the proposed classifier using augmented data has achieved quite competitive results for patients in both cohorts. In Table 8, we present a comparison between the median results obtained with the augmented model and the pooled estimation (95% CI) of the results of seven state-of-the-art articles that use machine learning to predict nocturnal hypoglycemia, which are directly obtained from the meta-analysis [35]. We include values of SEN, SP, and the $G_{mean}$.

## 5. Conclusions

The use of the technique described in this article sets a precedent as a solution to the problem of reduced data when developing machine learning models to predict real life medical events. It has been demonstrated that generative models are capable of expanding reduced data sets while preserving their intrinsic characteristics. As found in this study, data set augmentation provides researchers with the potential to realistically augment small and imbalanced data sets, leading to a general improvement in the predictive performance of machine learning models.

In addition, as the proposed models are able to represent the individualized glycemic response of each of the patients, they could be applied for sample anonymization purposes, which could contribute to more open and secure data sets in the face of data protection legislation. Furthermore, the application of this methodology with conditioning signals of insulin, meals, or exercise opens a path towards more realistic simulators that might be able to reduce the reality gap problem due to the improved ability to capture the whole human physiology that affects diabetic patients and which is unattainable using mathematical models.

## Figures and Tables

**Table 1:** Total number of instances for the ten patients of the Barcelona data set. Class 0 is defined as a sleep period without hypoglycemia and Class 1 is defined as a sleep period with hypoglycemia.

Patient ID	Total Instances	Class 1	Class 0
B1	85	6 (7%)	79 (93%)
B2	77	7 (9%)	70 (91%)
B3	94	22 (23%)	72 (77%)
B4	92	3 (3%)	89 (97%)
B5	82	6 (7%)	76 (93%)
B6	88	7 (8%)	81 (92%)
B7	103	31 (30%)	72 (70%)
B8	78	15 (19%)	63 (81%)
B9	110	13 (12%)	97 (88%)
B10	107	20 (19%)	87 (81%)

**Table 2:** Total number of instances for the six patients of the Ohio data set. Class 0 is defined as a sleep period without hypoglycemia and Class 1 is defined as a sleep period with hypoglycemia.

Patient ID	Total Instances	Class 1	Class 0
O1	34	1 (3%)	33 (97%)
O2	47	6 (13%)	41 (87%)
O3	40	3 (7%)	37 (93%)
O4	39	8 (21%)	31 (79%)
O5	52	3 (6%)	49 (94%)
O6	45	6 (13%)	39 (87%)

**Table 3:** Ranges of values where a patient’s blood glucose can be found.

Label	Range (mg/dL)
L2 Hypo	CGM < 54
L1 Hypo	54 ≤ CGM < 70
TIR	70 ≤ CGM < 180
Hyper	180 ≤ CGM

**Table 4:** Hardware specifications.

CPU	Intel Core i7-4770 CPU 3.40 GHz
Power Source	Corsair TX 750 M 750 Watt
GPU	Nvidia GeForce Titan Xp Pascal 12 GB GDDR5X
Mother Board	Asus Z87-A
RAM Memory	DDR3 16 GB

**Table 5:** Results obtained by comparing real and synthetic data for patients in the Barcelona cohort. All *p*-values have been accepted. as they pass the acceptance threshold of 0.05.

Patient		Hyper	TIR	L1 Hypo	L2 Hypo	Mean	JS	Variance	Z-Value	SD
B1	Real	48.80	45.21	3.74	2.26	187.20	0.23	−1.34	0.40	74.67
	Syn.	37.36	54.02	5.67	2.95	164.73	0.21	−1.37	0.59	58.59
B2	Real	28.79	61.94	5.01	4.26	147.95	0.19	−0.95	0.54	47.80
	Syn.	43.57	50.10	3.59	2.74	180.08	0.18	−1.56	0.72	56.69
B3	Real	35.08	56.69	5.24	2.99	158.78	0.21	−1.30	0.51	56.76
	Syn.	38.67	54.74	4.05	2.54	171.32	0.18	−1.74	0.77	54.35
B4	Real	44.72	47.80	4.07	3.40	175.15	0.22	−0.95	0.38	69.61
	Syn.	38.02	54.13	4.65	3.20	170.66	0.20	−1.25	0.54	66.39
B5	Real	39.52	54.94	4.29	1.24	163.54	0.19	−1.14	0.46	55.67
	Syn.	45.13	51.54	2.35	0.98	184.42	0.18	−1.42	0.60	59.73
B6	Real	51.37	41.11	3.21	4.31	185.68	0.22	−1.29	0.66	59.43
	Syn.	37.81	49.83	5.19	7.18	163.71	0.22	−1.29	0.66	59.43
B7	Real	35.40	50.53	7.21	6.85	153.87	0.25	−1.30	0.46	63.71
	Syn.	27.70	59.22	6.55	6.53	146.27	0.22	−1.41	0.54	54.17
B8	Real	32.84	56.30	7.06	3.80	154.30	0.23	−1.26	0.46	61.44
	Syn.	36.86	56.75	4.16	2.23	168.57	0.20	−1.56	0.54	63.66
B9	Real	46.01	45.59	4.85	3.55	176.19	0.24	−1.27	0.42	69.30
	Syn.	33.94	54.01	5.47	6.58	157.40	0.22	−1.56	0.66	57.23
B10	Real	36.59	47.59	6.30	9.52	160.35	0.28	−1.08	0.29	81.94
	Syn.	31.48	50.62	6.40	11.50	151.59	0.26	−1.40	0.44	74.35

**Table 6:** Results obtained by comparing real and synthetic data for patients in the Ohio cohort. All *p*-values have been accepted, as they pass the acceptance threshold of 0.05.

Patient		Hyper	TIR	L1 Hypo	L2 Hypo	Mean	JS	Variance	Z-Value	SD
O1	Real	39.27	56.92	2.74	1.07	167.12	0.21	−1.13	0.32	62.70
	Syn.	32.91	61.88	4.57	0.64	163.45	0.20	−1.92	0.50	59.84
O2	Real	25.72	72.13	1.85	0.30	148.04	0.17	−1.14	0.33	43.13
	Syn.	20.14	67.88	7.60	4.38	132.59	0.15	−1.90	0.85	34.86
O3	Real	60.30	38.60	1.03	0.07	195.18	0.14	−0.81	0.57	51.19
	Syn.	50.99	47.41	1.29	0.31	196.22	0.13	−2.12	1.07	48.59
O4	Real	25.13	67.62	5.12	2.14	144.20	0.21	−1.32	0.33	53.73
	Syn.	29.83	60.96	5.59	3.62	151.29	0.20	−1.49	0.54	56.82
O5	Real	38.61	60.66	0.56	0.17	167.99	0.15	−1.06	0.43	43.11
	Syn.	54.98	44.10	0.78	0.13	195.53	0.14	−1.07	0.68	49.08
O6	Real	31.16	65.06	3.19	0.59	153.16	0.19	−1.16	0.38	50.15
	Syn.	28.86	61.93	5.56	3.65	152.75	0.16	−1.43	0.88	41.71

**Table 7:** Results of a nocturnal hypoglycemia classifier trained with real and augmented data sets for patients of both Barcelona and Ohio cohorts.

Patient		ACC	SEN	SP	*G* ${}_{\mathrm{mean}}$	MCC
B1	Real	87.5	30.0	90.2	52.0	0.17
	Aug.	88.8	78.5	84.0	81.2	0.51
B2	Real	83.2	27.9	84.8	48.6	0.12
	Aug.	89.7	62.0	88.4	74.0	0.47
B3	Real	74.4	50.9	74.4	61.6	0.31
	Aug.	81.6	58.4	82.9	69.5	0.50
B5	Real	88.6	33.2	88.0	54.0	0.20
	Aug.	80.5	51.0	79.7	63.7	0.26
B6	Real	87.0	24.7	90.3	47.3	0.13
	Aug.	77.4	81.5	75.6	78.5	0.35
B7	Real	73.3	57.0	71.4	63.8	0.38
	Aug.	78.9	66.2	75.1	70.5	0.52
B8	Real	79.4	66.0	80.3	72.8	0.46
	Aug.	81.3	68.7	79.0	73.6	0.52
B9	Real	77.1	44.9	76.5	58.6	0.21
	Aug.	79.9	71.7	78.1	74.8	0.41
B10	Real	79.0	55.3	80.4	66.7	0.39
	Aug.	84.6	84.0	81.1	82.5	0.62
O2	Real	82.8	50.9	84.0	65.4	0.35
	Aug.	76.9	70.0	73.8	71.9	0.38
O4	Real	66.1	36.0	73.2	51.3	0.09
	Aug.	65.9	71.9	65.2	68.5	0.31
O6	Real	79.3	24.6	84.4	45.5	0.09
	Aug.	84.6	62.5	85.9	73.3	0.45
Med.	Real	79.4	40.4	82.2	56.3	0.21
	Aug.	80.9	69.3	79.3	73.5	0.46

**Table 8:** Comparison between state-of-the-art results and the obtained with the augmented data set, presented as medians and interquartile ranges.

	State-of-the-Art	Aug.
SEN	75 (70–80)	69 (62–74)
SP	65 (55–74)	79 (76–83)
$G_{mean}$	70 (62–77)	74 (70–76)

## Data Availability

Not applicable.

## Abbreviations

The following abbreviations are used in this manuscript:

T1DM	Type 1 Diabetes Mellitus
CGM	Continuous Glucose Monitor
Hyper	Hyperglycemia
L1 Hypo	Level 1 Hypoglycemia
L2 Hypo	Level 2 Hypoglycemia
AI	Artificial Intelligence
GAN	Generative Adversarial Network
EKG	Electrocardiogram
TIR	Time in Range
ReLU	Rectified Linear Unit
JS	Jensen–Shannon
SD	Standard Deviation
ACC	Accuracy
SEN	Sensitivity
SP	Specificity
MCC	Matthews Correlation Coefficient
$G_{mean}$	Geometric Mean
PLR	Positive Likehood Ratio
NLR	Negative Likehood Ratio

## References

[1] Felizardo V., Garcia N.M., Pombo N., Megdiche I. (2021). Data-based algorithms and models using diabetics real data for blood glucose and hypoglycaemia prediction—A systematic literature review. Artif. Intell. Med..

[2] Contreras I., Quirós C., Giménez M., Conget I., Vehi J. (2016). Profiling intra-patient type I diabetes behaviors. Comput. Methods Programs Biomed..

[3] Oviedo S., Contreras I., Quirós C., Giménez M., Conget I., Vehi J. (2019). Risk-based postprandial hypoglycemia forecasting using supervised learning. Int. J. Med. Inform..

[4] Oviedo S., Contreras I., Bertachi A., Quirós C., Giménez M., Conget I., Vehi J. (2019). Minimizing postprandial hypoglycemia in Type 1 diabetes patients using multiple insulin injections and capillary blood glucose self-monitoring with machine learning techniques. Comput. Methods Programs Biomed..

[5] Marling C., Bunescu R.C. (2018). The OhioT1DM Dataset For Blood Glucose Level Prediction. CEUR Workshop Proc..

[6] Kahn M. Diabetes. UCI Machine Learning Repository. https://archive-beta.ics.uci.edu/ml/datasets/diabetes.

[7] Contreras I., Vehi J. (2018). Artificial Intelligence for Diabetes Management and Decision Support: Literature Review. J. Med. Internet Res..

[8] Contreras I., Oviedo S., Vettoretti M., Visentin R., Vehí J. (2017). Personalized blood glucose prediction: A hybrid approach using grammatical evolution and physiological models. PLoS ONE.

[9] Woldaregay A.Z., Årsand E., Walderhaug S., Albers D., Mamykina L., Botsis T., Hartvigsen G. (2019). Data-driven modeling and prediction of blood glucose dynamics: Machine learning applications in type 1 diabetes. Artif. Intell. Med..

[10] Dalla Man C., Micheletto F., Lv D., Breton M., Kovatchev B., Cobelli C. (2014). The UVA/PADOVA type 1 diabetes simulator: New features. J. Diabetes Sci. Technol..

[11] Alkhalifah T., Wang H., Ovcharenko O. MLReal: Bridging the gap between training on synthetic data and real data applications in machine learning. Proceedings of the 82nd EAGE Annual Conference & Exhibition. European Association of Geoscientists & Engineers.

[12] Qin Z., Liu Z., Zhu P., Xue Y. (2020). A GAN-based image synthesis method for skin lesion classification. Comput. Methods Programs Biomed..

[13] Rashid H., Tanveer M.A., Aqeel Khan H. Skin Lesion Classification Using GAN based Data Augmentation. Proceedings of the 2019 41st Annual International Conference of the IEEE Engineering in Medicine and Biology Society (EMBC).

[14] Zhu F., Ye F., Fu Y., Liu Q., Shen B. (2019). Electrocardiogram generation with a bidirectional LSTM-CNN generative adversarial network. Sci. Rep..

[15] Piacentino E., Guarner A., Angulo C. (2021). Generating Synthetic ECGs Using GANs for Anonymizing Healthcare Data. Electronics.

[16] Yoon J., Drumright L.N., van der Schaar M. (2020). Anonymization Through Data Synthesis Using Generative Adversarial Networks (ADS-GAN). IEEE J. Biomed. Health Inform..

[17] Deng Y., Lu L., Aponte L., Angelidi A.M., Novak V., Karniadakis G.E., Mantzoros C.S. (2021). Deep transfer learning and data augmentation improve glucose levels prediction in type 2 diabetes patients. NPJ Digit. Med..

[18] De Paula F., Black D.M., Rossen J. (2017). Williams. Tratado de Endocrinología.

[19] Jameson J.L. (2017). Harrison’s Endocrinology.

[20] American Diabetes Association (2020). Glycemic Targets: Standards of Medical Care in Diabetes—2020. Diabetes Care.

[21] Bertachi A., Viñals C., Biagi L., Contreras I., Vehí J., Conget I., Giménez M. (2020). Prediction of Nocturnal Hypoglycemia in Adults with Type 1 Diabetes under Multiple Daily Injections Using Continuous Glucose Monitoring and Physical Activity Monitor. Sensors.

[22] Karras T., Aittala M., Laine S., Härkönen E., Hellsten J., Lehtinen J., Aila T. (2021). Alias-Free Generative Adversarial Networks. Adv. Neural Inf. Process. Syst..

[23] Wiese M., Knobloch R., Korn R., Kretschmer P. (2020). Quant GANs: Deep generation of financial time series. Quant. Financ..

[24] Goodfellow I., Pouget-Abadie J., Mirza M., Xu B., Warde-Farley D., Ozair S., Courville A., Bengio Y. (2014). Generative Adversarial Nets. Adv. Neural Inf. Process. Syst..

[25] Li K., Daniels J., Liu C., Herrero P., Georgiou P. (2020). Convolutional Recurrent Neural Networks for Glucose Prediction. IEEE J. Biomed. Health Inform..

[26] Fuglede B., Topsoe F. Jensen-Shannon divergence and Hilbert space embedding. Proceedings of the International Symposium on Information Theory.

[27] Zunino L., Olivares F., Ribeiro H.V., Rosso O.A. (2022). Permutation Jensen-Shannon distance: A versatile and fast symbolic tool for complex time-series analysis. Phys. Rev. E.

[28] Chollet F. Keras, 2015. GitHub. https://keras.io.

[29] Abadi M., Agarwal A., Barham P., Brevdo E., Chen Z., Citro C., Corrado S., Davis A., Dean J., Devin M. (2015). TensorFlow: Large-Scale Machine Learning on Heterogeneous Systems. tensorflow.org.

[30] McKinney W. Data Structures for Statistical Computing in Python. Proceedings of the 9th Python in Science Conference.

[31] Harris C.R., Millman K.J., Van Der Walt S.J., Gommers R., Virtanen P., Cournapeau D., Oliphant T.E. (2020). Array programming with NumPy. Nature.

[32] Pedregosa F., Varoquaux G., Gramfort A., Michel V., Thirion B., Grisel O., Duchesnay E. (2011). Scikit-learn: Machine Learning in Python. J. Mach. Learn. Res..

[33] Hunter J.D. (2007). Matplotlib: A 2D graphics environment. Comput. Sci. Eng..

[34] Mujahid O., Contreras I., Vehi J. (2021). Machine Learning Techniques for Hypoglycemia Prediction: Trends and Challenges. Sensors.

[35] Kodama S., Fujihara K., Shiozaki H., Horikawa C., Yamada M.H., Sato T., Sone H. (2021). Ability of Current Machine Learning Algorithms to Predict and Detect Hypoglycemia in Patients With Diabetes Mellitus: Meta-analysis. JMIR Diabetes.

